# Atypical frontotemporal cortical activity in first-episode adolescent-onset schizophrenia during verbal fluency task: A functional near-infrared spectroscopy study

**DOI:** 10.3389/fpsyt.2023.1126131

**Published:** 2023-03-08

**Authors:** Kun Zhang, Xingyue Jin, Yuqiong He, Shuxian Wu, Xilong Cui, Xueping Gao, Chunxiang Huang, Xuerong Luo

**Affiliations:** Department of Psychiatry, and National Clinical Research Center for Mental Disorders, The Second Xiangya Hospital of Central South University, Changsha, Hunan, China

**Keywords:** adolescent-onset schizophrenia, first-episode, functional near infrared spectroscopy (fNIRS), frontotemporal dysfunction, cognitive impairment

## Abstract

**Background:**

Frontotemporal cortex dysfunction has been found to be associated with cognitive impairment in patients with schizophrenia (SCZ). In patients with adolescent-onset SCZ, a more serious type of SCZ with poorer functional outcome, cognitive impairment appeared to occur at an early stage of the disease. However, the characteristics of frontotemporal cortex involvement in adolescent patients with cognitive impairment are still unclear. In the present study, we aimed to illustrate the frontotemporal hemodynamic response during a cognitive task in adolescents with first-episode SCZ.

**Methods:**

Adolescents with first-episode SCZ who were aged 12-17 and demographically matched healthy controls (HCs) were recruited. We used a 48-channel functional near-infrared spectroscopy (fNIRS) system to record the concentration of oxygenated hemoglobin (oxy-Hb) in the participants' frontotemporal area during a verbal fluency task (VFT) and analyzed its correlation with clinical characteristics.

**Results:**

Data from 36 adolescents with SCZ and 38 HCs were included in the analyses. Significant differences were found between patients with SCZ and HCs in 24 channels, mainly covering the dorsolateral prefrontal cortex, superior and middle temporal gyrus and frontopolar area. Adolescents with SCZ showed no increase of oxy-Hb concentration in most channels, while the VFT performance was comparable between the two groups. In SCZ, the intensity of activation was not associated with the severity of symptoms. Finally, receiver operating characteristic analysis indicated that the changes in oxy-Hb concentration could help distinguish the two groups.

**Conclusion:**

Adolescents with first-episode SCZ showed atypical cortical activity in the frontotemporal area during the VFT, and fNIRS features might be more sensitive indicators in cognitive assessment, indicating that the characteristic hemodynamic response pattern might be potential imaging biomarkers for this population.

## 1. Introduction

Schizophrenia (SCZ) is a major psychiatric disorder that seriously affects the functions and quality of life of patients. Apart from prominent positive and negative symptoms, different types of cognitive impairment also exist in most patients with SCZ. As cognitive impairment tends to be persistent even after the initial symptoms are relieved, it has been found that such impairment is significantly associated with the functional outcome of patients (1), indicating that it may be an important indicator of the prognosis of SCZ. Some researchers proposed that cognitive impairment is a core feature of SCZ (2).

Specifically, SCZ with an onset in adolescence is regarded as a more serious type of SCZ due to the poorer cognitive performance and functional outcomes in these patients (3). While adolescents with SCZ have similar symptoms to adult patients (4), they appeared to have more severe cognitive impairment. Compared with patients with adult-onset SCZ, adolescent patients may exhibit poorer performance in several cognitive domains (5), including executive function, psychomotor processing speed and verbal memory. As adolescence is a critical period for physical and mental development, cognitive impairment in the early stage of the disease (5) may explain the lower academic performance in individuals at a higher risk of psychosis (6). A 3-year follow-up study showed that only 15.8% of adolescents with SCZ were able to catch up with peers academically and up to 36.6% of them were unable to complete their education (7), indicating a heavy disease burden in this population. Studies on the underlying mechanism of cognitive impairment of adolescents with SCZ might help improve the functional outcome of patients as well as improve our understanding of this disorder.

Evidence has shown association between cognitive impairment in adult-onset SCZ and brain dysfunction, especially frontotemporal cortex dysfunction (8–11). Adolescence is a critical period of neurodevelopment, thus, abnormal brain function during this process may also be involved in the mechanism of cognitive impairment (6). However, considering that neurodevelopmental trajectories vary across different populations (12), results from studies on adult-onset SCZ might not be directly extrapolated to adolescent-onset SCZ. While functional magnetic resonance imaging (fMRI) studies of whole-brain resting-state functional connectivity (FC) determined the involvement of frontotemporal cortex in the pathology of adolescent SCZ (13), and abnormal FC in the left superior temporal gyrus was correlated with cognitive impairment in the diseased adolescents (14), few studies have focused on task related brain activity in adolescent SCZ; thus, the activity of the frontotemporal cortex during cognitive tasks is still unclear in adolescents with SCZ. Lately, functional near-infrared spectroscopy (fNIRS) has become a commonly used neuroimaging technology in the study of cerebral function. With its superiority in yielding data with good temporal and spatial resolution, it has been well accepted in studies involving cognitive task related hemodynamics. Moreover, the high safety and tolerance can enable its applicability in specific populations, including children and elderly people (8). Verbal fluency task (VFT) is widely employed in studies on the cognitive function of patients with SCZ (15); with the use of fNIRS, the hemodynamic response can be recorded simultaneously, providing direct cortical activation measurements during the cognitive tasks. Generally, during the VFT, patients with SCZ often show abnormal hemodynamic response patterns (8), decreased prefrontal cortex activity (16), and functional connectivity with lower intensity (17), as compared with healthy individuals. These findings have contributed to the understanding of the mechanism of cognitive impairment of patients with SCZ; however, whether the results can be extrapolated to adolescents still remains unknown.

In the present study, we aimed to illustrate the frontotemporal cortical activity patterns during a VFT in adolescents with SCZ. Based on previous studies, our hypothesis is that adolescents with first-episode SCZ show decreased VFT-related frontotemporal cortical activity compared with healthy adolescents. In order to evaluate the characteristics of cognitive impairment in the early stage of this disease, we only included patients with first-episode SCZ and explored whether the characteristics could help effectively distinguish patients from controls.

## 2. Methods

### 2.1. Participants

The participants were recruited from March 2021 to September 2022. SCZ patients were recruited from the Second Xiangya Hospital, China, with the inclusion criteria as follows: (1) meeting the diagnostic criteria of schizophrenia according to DSM-5; (2) with first-episode SCZ; (3) aged 12–17 years; (4) with an IQ above 70; and (5) right-handed. The exclusion criteria were (1) having received electroconvulsive therapy in the last 6 months; (2) comorbid with neurological diseases, other mental illnesses, serious physical diseases, cerebral trauma or other organic brain disorders. SCZ patients were diagnosed by child and adolescent psychiatrists with the intermediate professional title or above.

Healthy controls (HC) were recruited from the community with the following criteria: (1) aged 12–17 years; (2) with no mental illness according to the Schedule for Affective Disorders and Schizophrenia for School-Age Children-Present and Lifetime (KSADS-PL) interview (18); (3) with no family history of mental illnesses; (4) with an IQ above 70; and (5) right-handed. All the participants and their caregivers provided informed consent after fully informed.

### 2.2. Demographic statistics and clinical assessment

Demographic data including age, sex, and level of education were collected from all the participants. For patients with SCZ, the Positive and Negative Symptom Scale (PANSS) was used to assess symptoms of SCZ (19). This scale consists of 30 items in three subscales, i.e., positive symptoms, negative symptoms, and global psychopathology, with higher scores indicating higher severity of symptoms. The Chinese version of PANSS has good reliability and validity, with a standardized Cronbach α value of 0.8707 (20).

### 2.3. Verbal fluency task

All the participants underwent a VFT according to the programmed audio instruction on a computer. The 160-s block-designed task consisted of a 30-s pre-task baseline, a 60-s task, and a 70-s post-task baseline. For the pre- and post-task baseline periods, participants were instructed to repeat the numbers of 1, 2, 3, 4, and 5. During the task period, the participants were instructed to generate as many words including a given Chinese character as they could; referring to previous studies (21, 22), we chose three of the most commonly used Chinese characters for the task, namely “白 (white),” “天 (sky),” and “大 (big),” with each character phase lasting for 20-s. To ensure that the participants truly understood the task, they were allowed to practice with another character before the formal VFT started. During the task, the participants were seated in a comfortable chair and were instructed to avoid any major body movements. The number of valid words were recorded to reflect the task performance.

### 2.4. fNIRS measurements

**Figure 1:**
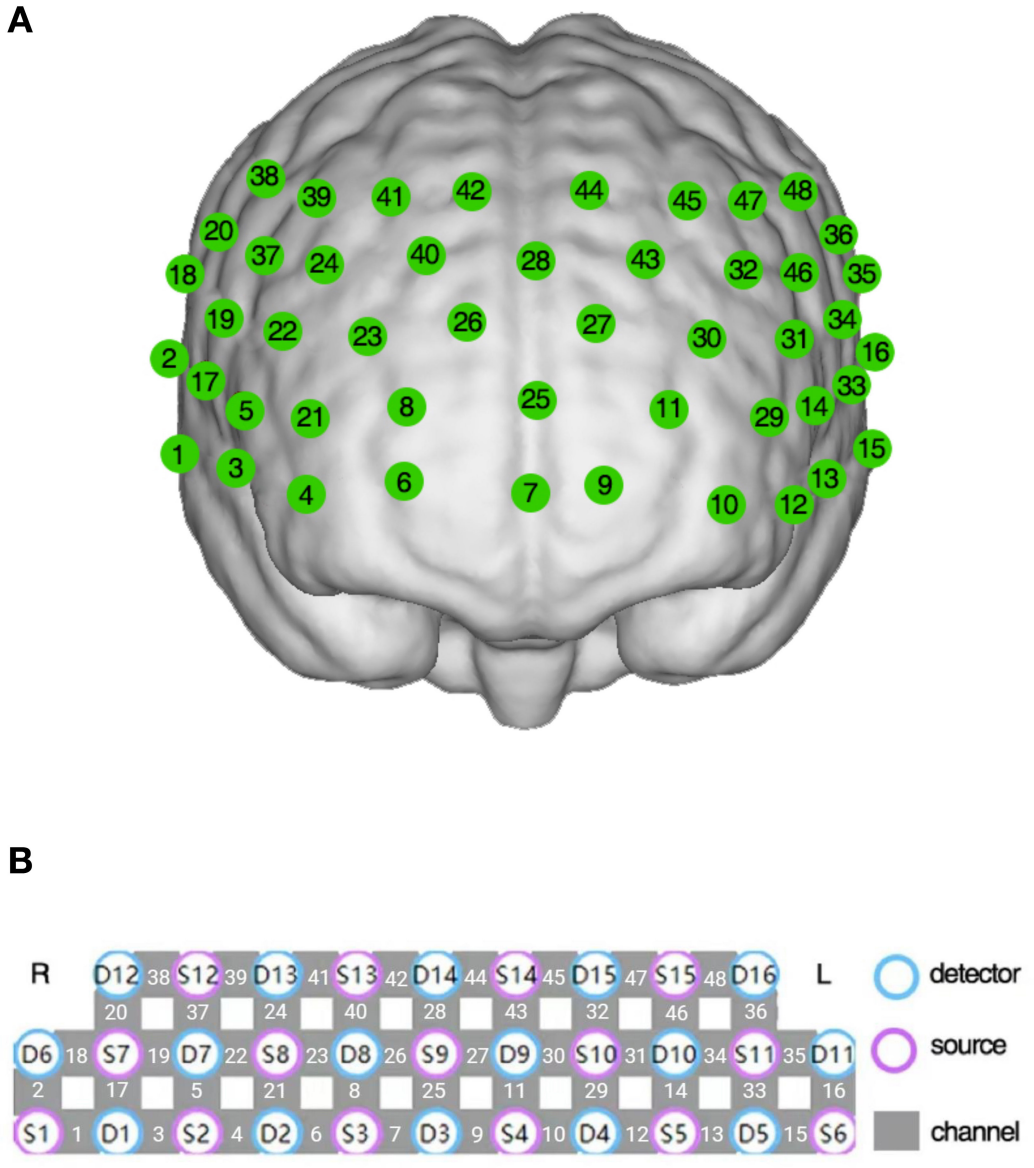
The 48-channel near-infrared spectroscopy system. **(A)** The location of each channel in the prefrontal and bilateral temporal region. **(B)** The arrangement of detectors, sources, and channels.

Changes in hemoglobin concentration during the VFT were measured using a 48-channel fNIRS system (NirSmart, Danyang Huichuang Medical Equipment Co., Ltd., China) according to the modified Beer-Lambert law (23). Two wavelengths of infrared light (730 nm and 850 nm) were used, with a sampling rate of 11 Hz. Neighboring infrared light sources and detectors were arranged at a distance of 3 cm, and the link between the neighboring source and detector was defined as the “channel.” The 48-channel system consisted of 15 source probes and 16 detector probes covering the prefrontal and bilateral temporal regions, the channels were located according to Brodmann area (24, 25) (Figures 1A, B).

### 2.5. fNIRS data processing

fNIRS data were processed using the NirSpark software package (HuiChuang, China). To remove systemic and physiological noise, we used a bandpass filter with a frequency range of 0.01–0.2 Hz to filter the raw data. The optical density was then translated to hemoglobin concentrations. In this study, we selected the concentration of oxygenated hemoglobin (oxy-Hb) as the target indicator of cortical activity, as the oxy-Hb signals have a higher signal-to-noise ratio (26) and the changes in oxy-Hb concentration is more sensitive for most mental diseases (27). Cortical activity was defined as relative oxy-Hb concentration change between the 60-s task period and the pre- and post-task baseline periods. To focus on task-specific signal changes, we carried out linear fitting on the data between the last 10 s of the pre-task baseline and the 5 s from the 40 to 45-s time points of the post-task baseline. Cortical activity was calculated for each participant during the VFT in all channels.

### 2.6. Statistical analysis

Shapiro-wilk test was used to test the normality of continuous variables. Independent two-sample *t*-test or Mann-Whitney U test was used to compare the inter-group difference in continuous variables, and Chi-square test was used to compare the categorical variables. The correlations between oxy-Hb concentration and clinical variables were analyzed using the Spearman correlation analysis. Receiver operating characteristic (ROC) analysis was used to assess the separation performance for oxy-Hb. All statistical analyses were performed using SPSS 22.0 (IBM, Inc., Chicago, Illinois), and figures were generated in R v3.6.3 (R Development Core Team 2020) and Rstudio v1.2.5033 (RStudio, Inc., 2019). *P*-values were adjusted with the Benjamini-Hochberg false discovery rate (FDR) procedure, with *p* < 0.05 (two-tailed) indicating that the difference was statistically significant.

## 3. Results

### 3.1. Demographic and clinical data

A total of 40 adolescents with schizophrenia (the SCZ group) and 38 healthy controls (the HC group) provided consent to participate in this study. Data from 36 adolescents with SCZ and 38 HCs were included in the final analyses as 4 patients with SCZ failed to complete the VFT. All the patients were at acute phase, and 17 adolescents with SCZ were medication-naive. No significant inter-group difference was found in age, sex ratio and level of education (Table 1).

**Table 1:** Demographics and clinical data.

	**SCZ (*n* = 36)**	**HC (*n* = 38)**	***t/x^2^/z***	***p* value**
Age (years)	14.56 ± 1.93	14.08 ± 1.26	1.249	0.217
Sex (male/female)	9/27	15/23	1.767	0.184
Education years	8.42 ± 1.76	7.97 ± 1.22	1.251	0.216
Duration of illness (months)	6.61 ± 6.60			
PANSS	79.09 ± 14.90			
Positive	20.40 ± 6.40			
Negative	20.31 ± 6.12			
General psychopathology	38.37 ± 8.00			
**Antipsychotic medication**				
Medication-naive (yes/no)	17/19			
Chlorpromazine dosage (mg)	260.51 ± 161.66			
VFT performance	7.53 ± 3.63	8.34 ± 3.99	−1.089	0.276

PANSS, Positive and Negative Syndrome Scale; SCZ, Schizophrenia; HC, Healthy control.

### 3.2. VFT performance and cortical activity

**Figure 2:**
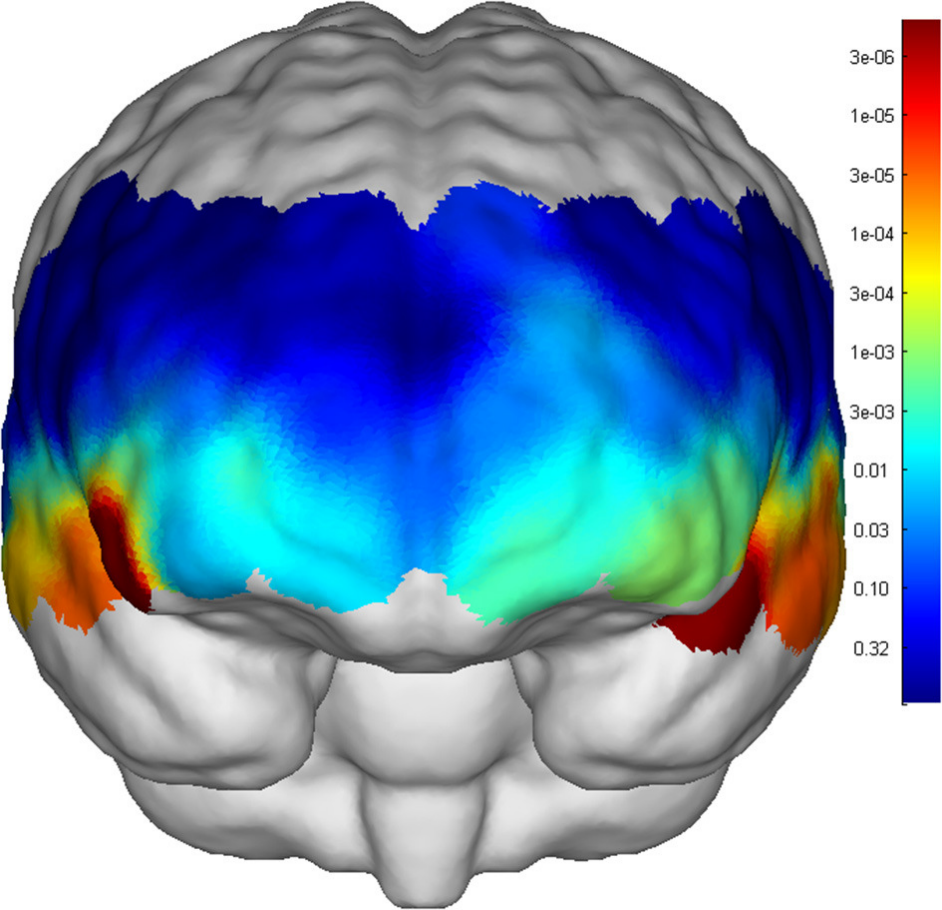
*p* values of each channel activation compared between SCZ and HC.

**Figure 3:**
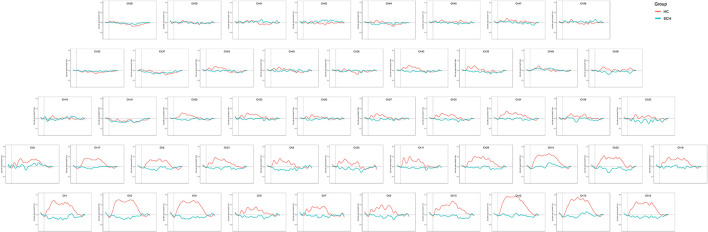
Waveforms of oxy-Hb concentration in each channel during the VFT.

The two groups of adolescents showed no significant difference in the number of words generated during the VFT [(7.53 ± 3.63) for the SCZ group vs. (8.34 ± 3.99) for HCs, *p* = 0.276]. Higher cortical activation, reflected by a greater increase in oxy-Hb concentration, was found in HCs compared with patients with SCZ in 24 channels: channels 1, 13 and 15 (mainly in the middle temporal gyrus), channels 6–11 (the frontopolar area), channels 16 and 33 (the superior temporal gyrus), channel 3 (the temporopolar area), channel 17 (the subcentral area), channels 5, 12, 14, 21, 29, and 31 (the triangularis Broca's area), and channels 4, 23, 27, 30 and 43 (the dorsolateral prefrontal cortex, DLPFC) (FDR *p* < 0.05, see Figure 2). Waveforms of oxy-Hb concentration change with time sequence in each channel are presented in Figure 3.

### 3.3. Correlations between VFT and clinical characteristics in SCZ

In adolescents with SCZ, the VFT performance was significantly correlated with the PANSS negative score (rho = −0.434, *p* = 0.009). Before FDR correction, positive correlation was found between the VFT performance and oxy-Hb concentration in channel 43 (DLPFC, rho = 0.442, *p* = 0.008); negative correlation was found between the PANSS total score and oxy-Hb concentration in channel 3 (temporopolar area, rho = −0.374, *p* = 0.029), between the PANSS positive score and oxy-Hb concentration in channel 4 (DLPFC, rho = −0.398, *p* = 0.020) and 13 (middle temporal gyrus, rho = −0.346, *p* = 0.045), between the PANSS general psychopathology score and oxy-Hb concentration in channel 4 (DLPFC, rho = −0.356, *p* = 0.039), and between the antipsychotic dosage and oxy-Hb concentration in channel 38 (pre-motor and supplementary motor cortex, rho = −0.586, *p* = 0.0389). However, these results did not pass the FDR correction.

### 3.4. Receiver operating characteristic (ROC) analysis

**Figure 4:**
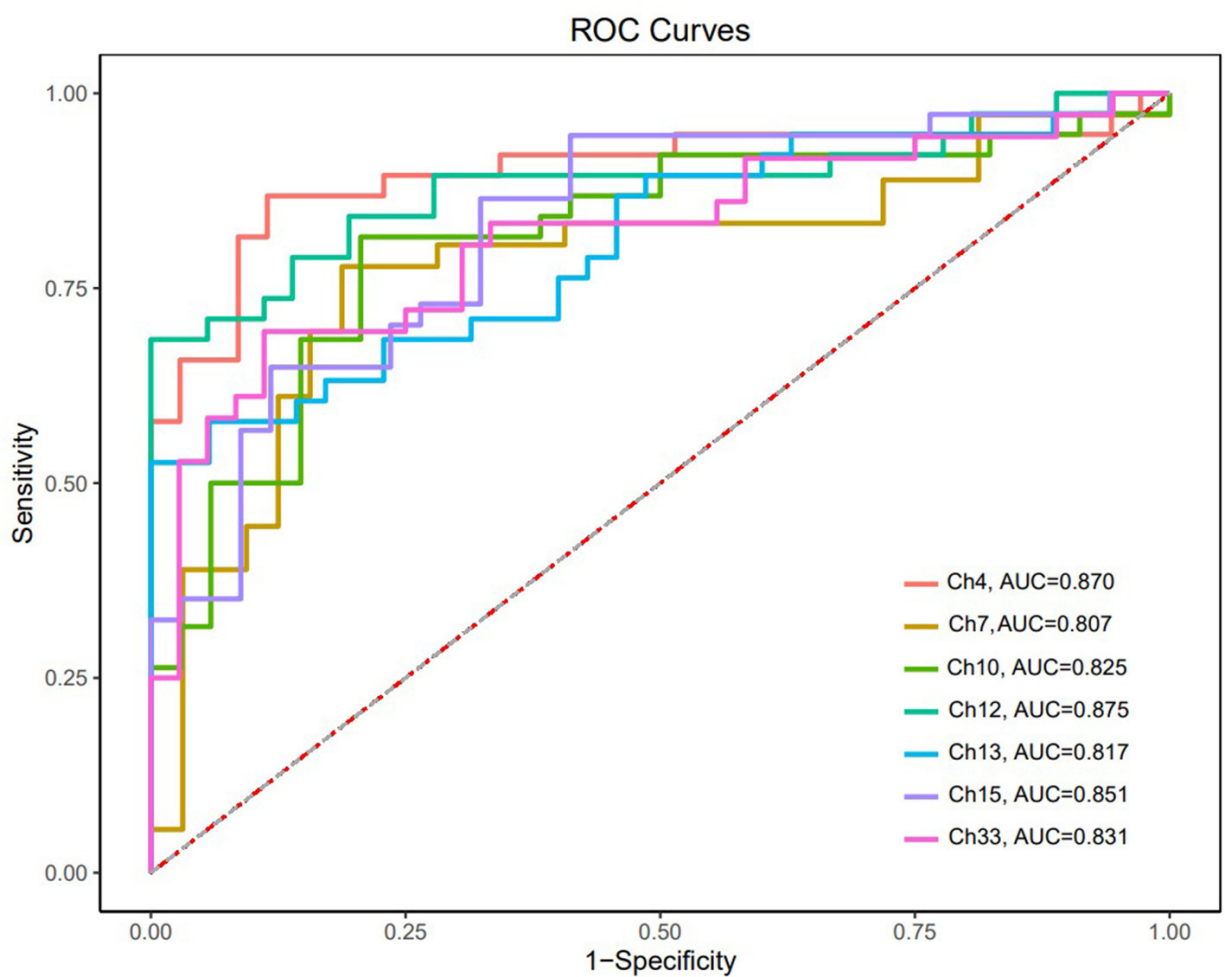
ROC curves of oxy-Hb changes in channels with AUC > 0.8.

The ROC curves were generated based on oxy-Hb changes in each channel. We calculated the areas under the curve (AUC) to assess their capability to distinguish between patients with SCZ and HCs. When the AUC was >0.8, the changes in oxy-Hb concentration in channel 4 (DLPFC), channels 7 and 10 (the frontopolar area), channel 12 (the triangularis Broca's area), channels 13 and 15 (the middle temporal gyrus), and channel 33 (the superior temporal gyrus) could help distinguish between the two groups effectively. The sensitivity and specificity of these channels are presented in Table 2, and the ROC curves are presented in Figure 4.

**Table 2:** Sensitivity and specificity of oxy-Hb change in channels with AUC > 0.8.

**Channel**	**AUC**	**Sensitivity**	**Specificity**
4	0.870	0.821	0.909
7	0.807	0.750	0.864
10	0.825	0.786	0.818
12	0.875	0.714	1.000
13	0.817	0.607	1.000
15	0.851	0.714	0.909
33	0.831	0.750	0.864

AUC, Area Under Curve.

## 4. Discussion

The present study found significant differences in VFT related frontotemporal cortical activity between adolescents with first-episode SCZ and their healthy counterparts, while the VFT performance was comparable between the two groups. We also found that the intensity of activation was not associated with the severity of symptoms, and that the impairment was similar across all patients with SCZ. To our knowledge, this is the first study on the pattern of frontotemporal cortical activity during the VFT in adolescents with first-episode SCZ.

### 4.1. Atypical cortical activity in SCZ

Adolescents with SCZ exhibited atypical cortical activity in 24 channels during VFT, covering half of the targeted areas. The greatest inter-group differences were found in channels 3, 4, 12, 13, 15, 33, involving the DLPFC, superior and middle temporal gyrus, and triangularis Broca's area. Channels 6–11 covers most of the frontopolar area where cortical activity also differed significantly between the two groups, suggesting abnormal frontotemporal function in the early stage of adolescent SCZ. A study revealed the association between lower activation in the frontopolar area and functional impairment in SCZ (28), indicating that the frontopolar function might be an important marker of functional outcome. The present study also found frontopolar abnormality in adolescents with SCZ, but whether this feature is associated with the functional outcome of adolescents with SCZ is worth further exploration.

Our findings are partly consistent with a former fMRI study showing lower activation in the middle frontal cortices in patients with acute psychosis and in remission (15). But interestingly, they had no remarkable finding in lateral prefrontal activation in patients with acute psychosis, which was inconsistent with the result of DLPFC dysfunction in the present study; their finding suggested that additional prefrontal activation may be involved in SCZ patients as neural compensation to maintain task performance. The VFT can reflect the executive function (29) and retrieval of information (30), considering the role of DLPFC in this process (9), the inconsistency in DLPFC dysfunction involvement might be associated with the diversity of neural compensation of DLPFC (31) in different SCZ population. The present study indicated that neural compensation might be uncommon among adolescents with first-episode SCZ.

Notably, no obvious increased frontotemporal activity was observed in adolescents with SCZ. This is inconsistent with a former VFT study on adult patients with first-episode SCZ (32); they found that although oxy-Hb concentration increase in the patient group was lower than in HC, the patients showed significantly increased frontotemporal activities during the VFT in most channels. Similar frontotemporal activation pattern was also found in adult patients with chronic SCZ (22, 33, 34). However, in the present study, the patient group showed no obvious increase in oxy-Hb concentration after the 60-s task period started, and even a decrease of oxy-Hb concentration was found in some channels (Figure 3). The broad inhibition of hemodynamic response in the frontotemporal area in adolescents with SCZ demonstrated an atypical cortical activity pattern which might be specific to adolescent patients. Existing evidence suggested abnormal neurodevelopment in adolescents with SCZ. In children and adolescents with SCZ, decreased gray matter volume was found mainly in the frontotemporal cortex (35). In a resting-state fMRI study, the researchers attributed the abnormal anatomical FC pattern in DLPFC to functional overpruning in the neurodevelopment process (13). The present study might provide support for the neurodevelopmental disorder theory of SCZ and indicated that adolescent-onset SCZ might be a more serious type of SCZ.

In addition, we found no significant correlation between the intensity of activation and the PANSS score or other clinical characteristics, indicating that the change in oxy-Hb concentration during VFT was not sensitive to SCZ symptoms.

### 4.2. VFT performance

We found that the adolescents with SCZ performed equally well in generating words compared with HC. Concerning the VFT performance is associated with the difficulty of task (36), the phonological fluency task used might be equally difficult for the adolescents with first-episode SCZ and HCs. Some researchers proposed that the category fluency task (CFT) might be superior for the evaluation of Chinese-speaking patients with psychiatric disorders (34). Thus, further studies on potential inter-task effects in adolescents are still needed. Meanwhile, although there was no significant difference in the VFT performance between the two groups, the patients' atypical cortical activity pattern suggested that the functional changes in the frontotemporal area occurred prior to cognitive impairment and that fNIRS features might be more sensitive in cognitive assessment. Moreover, in SCZ group, no significant correlation was found between the VFT performance and oxy-Hb concentration, indicating that, decreased frontotemporal cortical activity was common among adolescents with first-episode SCZ and may be associated with neural inefficiency or abnormalities in neurovascular coupling rather than lack of motivation for cognitive task (27).

In adolescents with SCZ, the VFT performance was negatively correlated with the PANSS negative score, i.e., patients with more severe negative symptoms came up with fewer words in the VFT, indicating an association between negative symptoms and cognitive impairment (37, 38). According to prior studies, the brain regions associated with negative symptoms and those associated with cognitive impairments overlapped mainly in the frontotemporal cortex (9, 39).

### 4.3. ROC analysis

The ROC analysis found that the change in oxy-Hb concentration in 7 channels had a good sensitivity and specificity for distinguishing adolescents with SCZ from their healthy counterparts, with a high sensitivity of 0.821 and specificity of 0.909 obtained in channel 4. In addition to previous findings that neurological features based on the frontotemporal cortex are potential biomarkers for SCZ (40), our study suggested that oxy-Hb changes during the VFT might be a promising imaging biomarker for adolescents with SCZ; however, large-scale studies are needed to verify our findings.

## 5. Limitations

There are still some limitations to this study. First, some of the participants were on antipsychotic medications. Although we found no significant correlation between antipsychotic dosage and oxy-Hb changes, the potential influence of medications might need to be investigated in further studies involving all medication-naive adolescents with SCZ. Second, the results of ROC analysis have not been extrapolated to external samples, thus, the oxy-Hb change during VFT in distinguishing patients with SCZ from HCs needs to be further verified. Last, we focused on adolescents with first-episode SCZ in this study, future studies involving adolescents in the chronic phase of SCZ, or a longer duration of illness are warranted.

## 6. Conclusion

Adolescents with SCZ showed atypical cortical activity in the frontotemporal area during the VFT, and the characteristic hemodynamic response pattern may be a potential imaging biomarker for this population.

## Data availability statement

The raw data supporting the conclusions of this article will be made available by the authors, without undue reservation.

## Ethics statement

The studies involving human participants were reviewed and approved by the Ethics Committee of Second Xiangya Hospital. Written informed consent to participate in this study was provided by the participants' legal guardian/next of kin.

## Author contributions

XL and KZ were responsible for the study design. KZ, XJ, and YH were responsible for recruiting the participants. KZ, SW, and XJ were involved in statistical analysis. KZ, XC, and YH were involved in manuscript preparation and drafting the paper. CH and XG were involved in editing and revising the manuscript. XL was responsible for the critical revision of the manuscript. All authors contributed to the article and approved the submitted version.
